# Fms-Like Tyrosine Kinase 3-Independent Dendritic Cells Are Major Mediators of Th2 Immune Responses in Allergen-Induced Asthmatic Mice

**DOI:** 10.3390/ijms21249508

**Published:** 2020-12-14

**Authors:** Sang Chul Park, Dahee Shim, Hongmin Kim, Yeeun Bak, Da Yeon Choi, Joo-Heon Yoon, Chang-Hoon Kim, Sung Jae Shin

**Affiliations:** 1 Department of Otorhinolaryngology-Head and Neck Surgery, Kangnam Sacred Heart Hospital, Hallym University College of Medicine, Seoul 07441, Korea; newliebe@hanmail.net; 2 Department of Microbiology, Yonsei University College of Medicine, Seoul 03722, Korea; dahee0219@yuhs.ac (D.S.); goldhm@yuhs.ac (H.K.); yeeun_bak@yuhs.ac (Y.B.); 3 Brain Korea 21 Program for Leading Universities and Students (PLUS) Project for Medical Science, Yonsei University College of Medicine, Seoul 03722, Korea; 4 Hallym University Industry-Academic Cooperation Foundation, Chuncheon 24252, Korea; dy8164@naver.com; 5 Department of Otorhinolaryngology, Yonsei University College of Medicine, Seoul 03722, Korea; jhyoon@yuhs.ac; 6 Global Research Laboratory for Allergic Airway Diseases, Yonsei University College of Medicine, Seoul 03722, Korea; 7 The Airway Mucus Institute, Yonsei University College of Medicine, Seoul 03722, Korea; 8 Department of Microbiology, Institute for Immunology and Immunological Diseases, Yonsei University College of Medicine, Seoul 03722, Korea

**Keywords:** allergic asthma, dendritic cells, Fms-like tyrosine kinase 3, murine model, OX40 ligand, Th2 immune responses

## Abstract

Dendritic cells (DCs) are the main mediators of Th2 immune responses in allergic asthma, and Fms-like tyrosine kinase 3 ligand (Flt3L) is an important growth factor for the development and homeostasis of DCs. This study identified the DC populations that primarily cause the initiation and development of allergic lung inflammation using Fms-like tyrosine kinase 3 (Flt3) knockout (KO) mice with allergen-induced allergic asthma. We observed type 2 allergic lung inflammation with goblet cell hyperplasia in Flt3 KO mice, despite a significant reduction in total DCs, particularly CD103^+^ DCs, which was barely detected. In addition, bone marrow-derived dendritic cells (BMDCs) from Flt3 KO mice directed Th2 immune responses in vitro, and the adoptive transfer of these BMDCs exacerbated allergic asthma with more marked Th2 responses than that of BMDCs from wild-type (WT) mice. Furthermore, we found that Flt3L regulated the in vitro expression of OX40 ligand (OX40L) in DCs, which is correlated with DC phenotype in in vivo models. In conclusion, we revealed that Flt3-independent CD11b^+^ DCs direct Th2 responses with the elevated OX40L and are the primary cause of allergic asthma. Our findings suggest that Flt3 is required to control type 2 allergic inflammation.

## 1. Introduction

Allergic asthma is a complex chronic inflammation of the airways characterized by airway hyperresponsiveness (AHR), goblet cell hyperplasia, epithelial hypertrophy, and mucus hypersecretion [1,2,3]. Numerous inflammatory cells are responsible for the pathophysiological changes of asthma, eventually leading to disruption of the balanced T cell responses [4,5,6]. Among them, dendritic cells (DCs) are the most specialized antigen-presenting cells that mediate the innate and adaptive immune responses [2,7,8]. Typically, DCs are activated by inhaled allergens and migrate to the draining mediastinal lymph nodes, where they interact with T cells to induce Th2 immune responses [8,9,10,11]. 

DCs are a heterogeneous population, and various DC types contribute to the aggravation or suppression of allergic inflammation [9,12,13,14,15]. They originate from hematopoietic stem cells in the bone marrow (BM), which differentiate into macrophage DC precursors and then to common dendritic cell precursors (CDPs) and common monocyte progenitors (cMoPs). CDPs develop into pre-conventional DCs (pre-cDCs) and plasmacytoid DCs (pDCs), whereas cMoPs become monocytes and monocyte-derived DCs (moDCs) [16,17]. Pre-cDCs consist of pre-cDC1s and pre-cDC2s, which are committed to the conventional DCs type 1 (cDC1s) and conventional DCs type 2 (cDC2s) lineages, respectively. cDC1s express CD8α in lymphoid organs but CD103 in non-lymphoid tissues, whereas cDC2s express CD11b. moDCs also express CD11b but are recruited only under inflammatory conditions [18]. The development of CDPs is regulated by the growth factor Fms-like tyrosine kinase 3 ligand (Flt3L) and its receptor Fms-like tyrosine kinase 3 (Flt3) [13,18]. Flt3L strongly expands cDCs and pDCs in vivo [19] and can be used to generate all functional DC subsets in vitro [20]. In contrast, mice lacking Flt3L display a severe deficiency in DCs [21]. Notably, cDC1s are preferentially affected by Flt3L levels [22].

Studies on the effects of Flt3L in various diseases have shown contradictory results, typically in experiments using Flt3 treatment and ligand knockout (KO) or receptor KO. For example, in rheumatoid arthritis, Flt3 showed conflicting effects on inflammation. The administration of Flt3L reduced the severity of arthritis by inducing the activity of regulatory T cells (Tregs) [23]; however, Fl3tL-deficient mice were protected from arthritis by decreased T cell activation [24]. Additionally, in colitis models, Flt3L treatment preferentially led to the expansion of CD103^+^ DCs and Tregs, alleviating Crohn’s-like murine ileitis [25], whereas in another study, CD103^+^ DCs favored the emergence of interferon-γ (IFN-γ)-producing CD4^+^ T cells, thereby abrogating tolerogenic properties [26]. Interestingly, in the lung, Flt3L treatment reversed AHR to methacholine and attenuated airway inflammation in an asthma mouse model [27]. This was attributed to the increase in CD4^+^CD25^+^Foxp3^+^ICOS^+^IL-10^+^ Tregs in the asthmatic lung [28]. However, Flt3-deficient mice have not been investigated in detail using asthma models.

Currently, which subset of DC populations exacerbates the development of allergic inflammation is controversial. Human blood dendritic cell antigen 3 (BDCA-3)^+^ DCs, corresponding to CD103^+^ DCs in mice, are known to induce the activity of peripheral Tregs [29]. Additionally, our previous studies on the expression of the DC subset in human nasal mucosa revealed that BDCA-3^+^ DCs are inversely proportional to disease severity, serum eosinophils, and immunoglobulin (Ig) E levels [30]. However, BDCA-3 expression on DCs was increased in atopic patients compared to that in control subjects [31], and increased numbers of BDCA-3^+^ DCs were detected in the bronchoalveolar lavage fluid (BALF) of patients with allergy [32]. 

Therefore, we investigated which DC populations (CD103^+^ DCs versus CD11b^+^ DCs) are crucial for the initiation and development of allergic asthma in Flt3 KO mice. We confirmed our results through in vitro and in vivo studies. 

## 2. Results

### 2.1. Flt3 KO Mice with Allergen-Induced Asthma Exhibit Exacerbated Goblet Cell Hyperplasia and Elevated Serum Allergen-Specific IgE Levels

**Figure 1:**
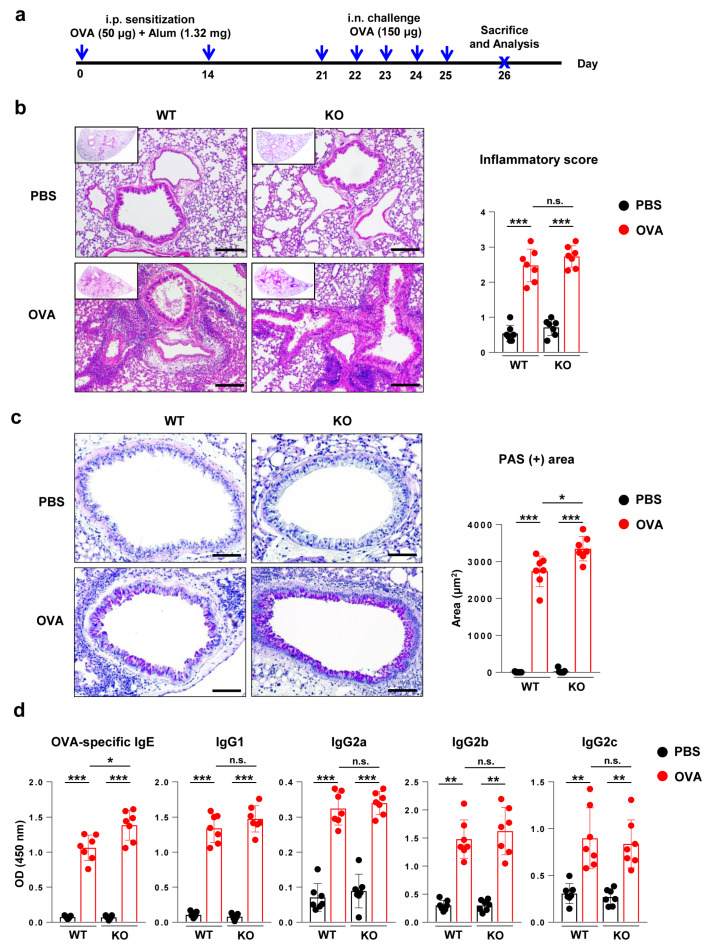
Comparison of disease phenotype between wild-type (WT) and Fms-like tyrosine kinase 3 (Flt3) knockout (KO) mice with ovalbumin (OVA)-induced asthma. (**a**) Experimental scheme and protocol for a mouse model of OVA-induced allergic asthma. Arrows indicate the time points of procedures or sacrifice. i.p., intraperitoneal; i.n., intranasal. (**b**) Representative hematoxylin and eosin-stained sections (original magnification: ×100, magnified image of the ×10 figure in the left upper quadrant) and inflammatory score based on lung histology (scale bar: 100 μm). (**c**) Representative periodic acid-Schiff (PAS)-stained section (original magnification: ×200) and PAS-positive areas based on lung histology (scale bar: 50 μm). (**d**) Serum OVA-specific immunoglobulin (Ig)E, IgG1, IgG2a, IgG2b, and IgG2c were measured using ELISA. Data are representative of four independent experiments with seven mice/group in each experiment. The results are expressed as the mean ± standard deviation. The significance of differences was analyzed using an unpaired Student’s *t*-test. * *p* < 0.05, ** *p* < 0.01, *** *p* < 0.001.

First, we examined the phenotype of asthma in the ovalbumin (OVA)-induced asthma model of wild-type (WT) and Flt3 KO mice (Figure 1a). Peribronchial, perivascular, and parenchymal infiltration of inflammatory cells was observed in the OVA-challenged groups, which was comparable between Flt3 KO mice and WT mice (*p* = 0.281; Figure 1b). Goblet cells were observed in the OVA-challenged groups. In OVA-treated Flt3 KO mice, goblet cell hyperplasia was increased compared to that in OVA-treated WT mice, which was confirmed by measuring periodic acid-Schiff (PAS)-positive areas (*p* < 0.05; Figure 1c). In the OVA-challenged groups, the levels of OVA-specific immunoglobulin (Ig) E and IgG (G1, G2a, G2b, and G2c) were elevated compared to those in the phosphate-buffered saline (PBS)-challenged groups. Notably, OVA-specific IgE levels were elevated in OVA-treated Flt3 KO mice compared to those in OVA-treated WT mice (*p* < 0.05; Figure 1d).

### 2.2. Flt3 KO Mice with Asthma Show Decrease of DCs in BALF without Alterations in the Total Immune Cell Populations

**Figure 2:**
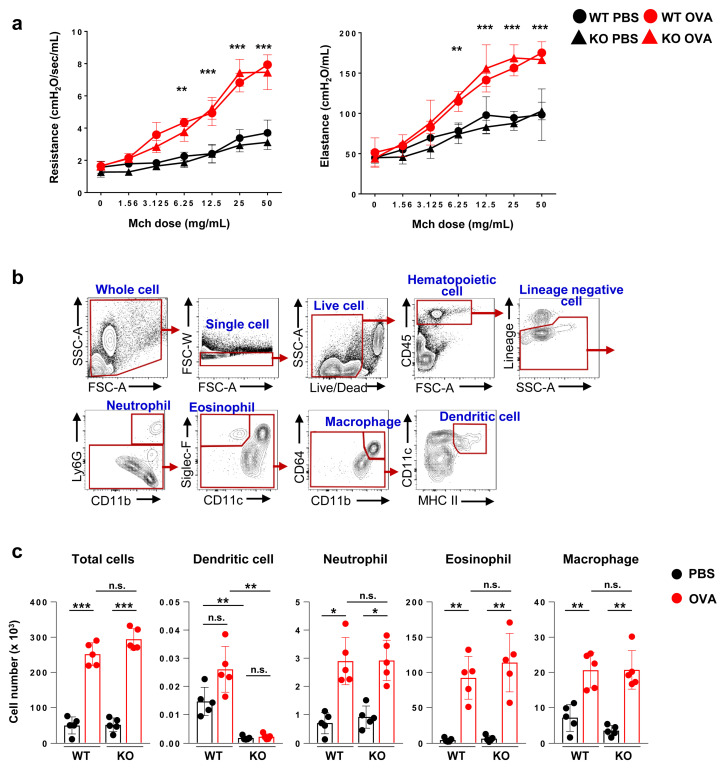
Comparative evaluation of airway hyperresponsiveness (AHR) and analysis of cellular composition in bronchoalveolar lavage fluid (BALF). (**a**) Airway resistance and airway elastance after methacholine challenge. (**b**) Representative flow cytometry plots for immune cells including dendritic cells in BALF. (**c**) Total cell count and differential cell count of BALF. Data are representative of three independent experiments with five mice/group in each experiment. The results are expressed as the mean ± standard deviation. The significance of differences was analyzed using an unpaired Student’s *t*-test. * *p* < 0.05, ** *p* < 0.01, *** *p* < 0.001.

Next, we examined the AHR, which is another key feature of allergic asthma. Airway resistance and elastance after methacholine exposure were increased in the OVA-challenged groups compared to those in the PBS-challenged groups. AHR was not significantly different between OVA-treated WT mice and OVA-treated Flt3 KO mice (Figure 2a).

We next investigated the immune cell population of the BALF and lung in Flt3 KO mice compared to that in WT mice before and after OVA challenge (Figure 2b). Total and differential cell counts from the BALF were higher in the OVA-challenged groups than in the PBS-challenged groups. In both groups, the number of DCs was significantly decreased (*p* < 0.01) in Flt3 KO mice compared to that in WT mice. Interestingly, other immune cells were comparable between OVA-treated WT mice and OVA-treated Flt3 KO mice (Figure 2c).

### 2.3. Flt3 KO Mice with Asthma Exhibit Decreased the Population of Total DCs, Particularly CD103^+^ DCs, Whereas They Show a Relative Expansion of CD11b^+^ DCs in the Lung

**Figure 3:**
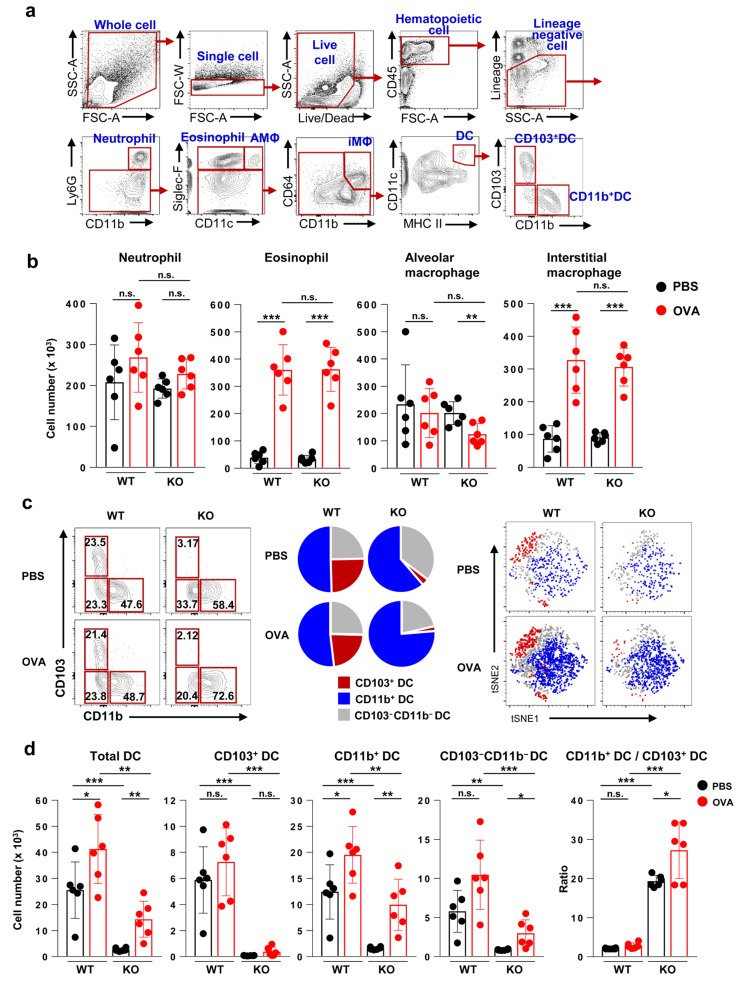
Analysis of myeloid cell compositions and characteristics of dendritic cell (DC) subsets in the lungs of wild-type (WT) and Flt3 knockout (KO) mice. (**a**) Representative flow cytometry plots for immune cells including dendritic cells in lung. AMΦ, alveolar macrophage; iMΦ, interstitial macrophage; DC, dendritic cell. (**b**) Population of myeloid cells in the lung. (**c**) Proportion of each DC subset among total DCs in the lung presented as representative plots, pie charts, and the t-distributed stochastic neighbor embedding (t-SNE) map. (**d**) Quantification of total and subsets of DCs and the ratio of CD11b^+^ DCs to CD103^+^ DCs in lung. Data are representative of three independent experiments with six mice/group in each experiment. The results are expressed as the mean ± standard deviation. The significance of differences was analyzed using an unpaired Student’s *t*-test. * *p* < 0.05, ** *p* < 0.01, *** *p* < 0.001.

Then, we compared the immune cell populations of the lung between Flt3 KO mice and WT mice. The OVA-challenged groups exhibited higher eosinophil and interstitial macrophage counts than the PBS-challenged groups. However, there was no significant difference in immune cell populations between OVA-treated WT mice and OVA-treated Flt3 KO mice (Figure 3a). Therefore, we focused on analyzing DCs and determined the subtype of DCs dominantly involved in allergic inflammation.

In the lung, total DCs and CD11b^+^ DCs were significantly decreased in Flt3 KO mice compared to those in WT mice (*p* < 0.001); notably, CD103^+^ DCs were nearly absent from Flt3 KO mice (*p* < 0.001). OVA challenge induced the recruitment of DCs, particularly CD11b^+^ DCs, which was more apparent in Flt3 KO mice than in WT mice. The ratio of CD11b^+^ DCs to CD103^+^ DCs was markedly elevated in OVA-treated Flt3 KO mice compared to that in OVA-treated WT mice (*p* < 0.001) (Figure 3b).

### 2.4. Flt3 KO Mice with Asthma Exhibit a Shift toward Th2 Immune Responses in the Lung and Spleen 

**Figure 4:**
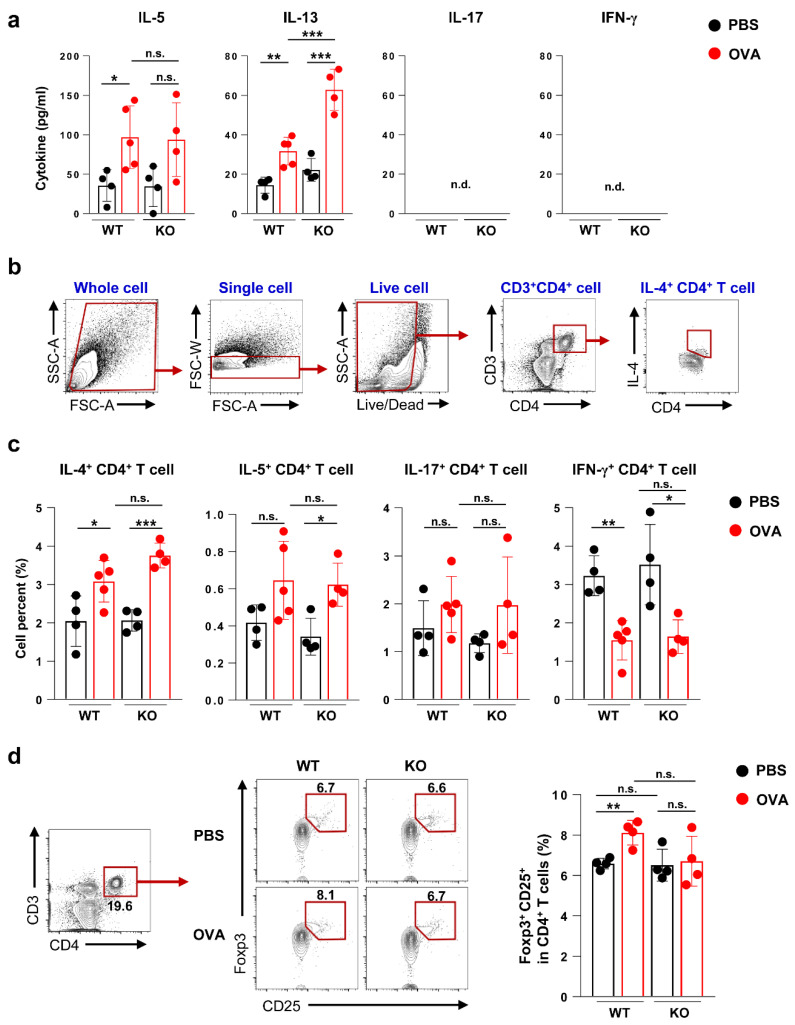
Cytokine profiles and analysis of T cell subpopulations in lungs of wild-type (WT) and Flt3 knockout (KO) mice. (**a**) Cytokines were measured using ex vivo recall assays with ovalbumin (OVA) protein (10 μg/mL) in isolated lung cells. (**b**) Representative flow cytometry plots for the examination of intracellular cytokines of CD4^+^ T cells in the lung. (**c**) Intracellular levels of interferon-γ (IFN-γ), interleukin (IL)-4, IL-5, and IL-17 of CD4^+^ T cells in the lung were measured using flow cytometry after stimulation with OVA (10 μg/mL) for 2 h with GolgiStop. (**d**) CD4^+^CD25^+^Foxp3^+^ regulatory T cells (Treg) were gated from CD4^+^ T cells using flow cytometry. Data are representative of three independent experiments with four or five mice/group in each experiment. The results are expressed as the mean ± standard deviation. The significance of differences was analyzed using an unpaired Student’s *t*-test. * *p* < 0.05, ** *p* < 0.01, *** *p* < 0.001. n.d., not detected.

Then, we investigated the cytokine expression and immune responses of T cells to examine the effect of DCs. Cytokine expression was evaluated in OVA-restimulated lung and spleen cells. The expression of interleukin (IL)-13 was significantly increased in OVA-treated Flt3 KO mice compared to that in OVA-treated WT mice in both the lung (*p* < 0.001; Figure 4a) and spleen (*p* < 0.05; Supplementary Figure S1a), whereas IL-5 (lung), IFN-γ, and IL-17 (spleen) did not significantly differ between OVA-treated Flt3 KO mice and OVA-treated WT mice (Figure 4a and Supplementary Figure S1a). The expression of intracellular cytokines of CD4^+^ T cells in the lung was also measured (Figure 4b and Supplementary Figure S1b). In the OVA-challenged groups, IL-4^+^ CD4^+^ T cells, IL-5^+^ CD4^+^ T cells, and IL-17^+^ CD4^+^ T cells tended to increase, whereas IFN-γ^+^ CD4^+^ T cells decreased compared to those in the PBS-challenged groups. The population of IL-4^+^ CD4^+^ T cells tended to increase in OVA-treated Flt3 KO mice than in OVA-treated WT mice (*p* = 0.066; Figure 4c).

Based on Treg analysis, the baseline Treg population was comparable between the lungs of WT and Flt3 KO mice. In WT mice, OVA challenge induced Treg expansion to control inflammation, which was not observed in Flt3 KO mice (Figure 4d). 

Taken together, these data (Figure 1, Figure 2, Figure 3 and Figure 4) show that allergic airway inflammation was sufficiently induced in Flt3 KO mice, even though DCs, and particularly CD103^+^ DCs, were deficient. Moreover, immune responses (serum OVA-specific IgE and IL-13 in the lung and spleen) exhibited a more pronounced shift toward Th2 responses in OVA-treated Flt3 KO mice compared to those in OVA-treated WT mice.

### 2.5. Bone Marrow-Derived Dendritic Cells (BMDCs) Generated from Flt3 KO Mice More Potently Induce Th2 Responses Than Those from WT Mice during In Vitro Co-Culture with OVA-Specific Transgenic CD4^+^ T Cells

**Figure 5:**
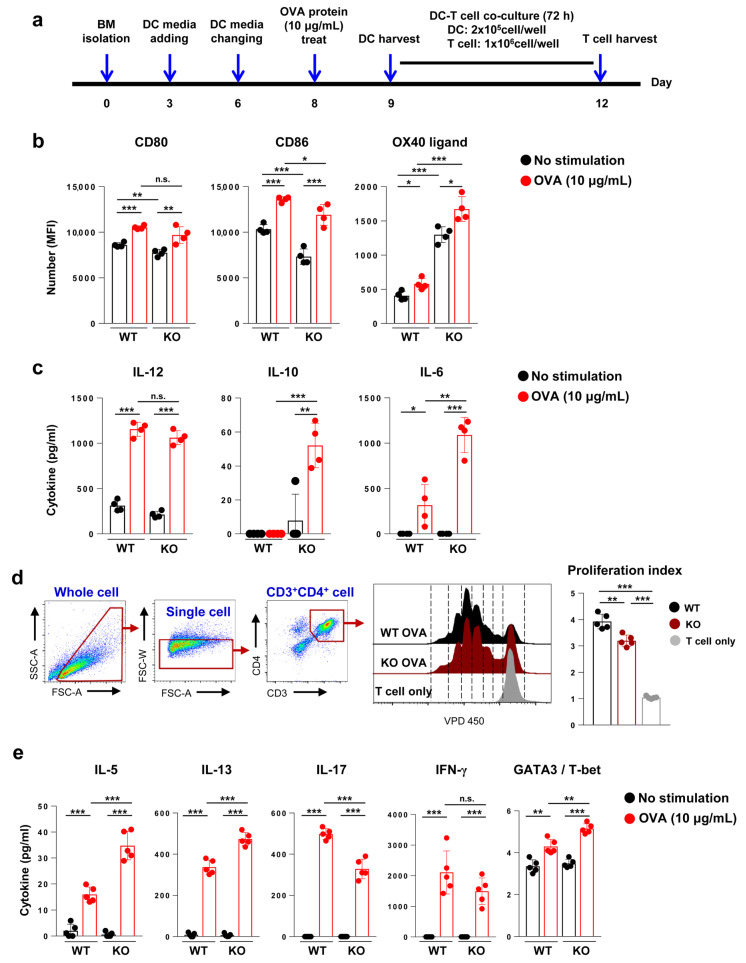
Comparison of Th2-inducing ability of bone marrow-derived dendritic cells (BMDCs) from wild-type (WT) and Flt3 knockout (KO) mice by in vitro co-culture with OT-II CD4^+^ T cells. (**a**) Experimental schematic protocol of BMDC generation and co-culture with CD4^+^ T cells from the spleens of ovalbumin (OVA)-specific OT-II Tg mice. Arrows indicate the time points of procedures. (**b**) The expression of CD80, CD86, and OX40 ligand was measured using flow cytometry. (**c**) Comparison of cytokines produced by BMDCs. (**d**) T cell proliferation was assessed in CD4^+^ T cells co-cultured with BMDCs. The proliferation rate is shown in the histogram and as the proliferation index. (**e**) Cytokines produced by CD4^+^ T cells cultured with BMDCs and T cell transcription factors expressed as the GATA3/T-bet ratio. Data are representative of three independent experiments with four or five mice/group in each experiment. The results are expressed as the mean ± standard deviation. The significance of differences was analyzed using an unpaired Student’s *t*-test. * *p* < 0.05, ** *p* < 0.01, *** *p* < 0.001.

Since DCs—independent of the Flt3 signal—were sufficient to induce asthma upon OVA challenge in Flt3 KO mice, we hypothesized that Flt3-independent DCs (DCs from Flt3 KO mice) could better induce type 2 immune responses than Flt3-dependent DCs (DCs from WT mice). To verify this in vitro, bone marrow-derived dendritic cells (BMDCs) from WT and Flt3 KO mice were obtained as shown in Figure 5a. BMDCs generated with granulocyte-macrophage colony-stimulating factor (GM-CSF) were predominantly positive for CD11b, which was consistent with a previous report (Supplementary Figure S2a) [33]. The number of harvested BMDCs from Flt3 KO mice was two-thirds that obtained from WT mice (1.64 × 10^8^ cells vs. 2.43 × 10^8^ cells, respectively) (Supplementary Figure S2b).

To compare DC functions between WT and Flt3 KO mice, we measured the expression levels of DC surface molecules related to T cell activation. The representative plots of each marker in WT and Flt3 KO mice are shown in Supplementary Figure S2c. The expressions of CD80 and CD86 were lower in BMDCs from Flt3 KO mice than in those from WT mice. However, OX40 ligand (OX40L), which is important for driving Th2 responses [34,35], was upregulated in BMDCs from Flt3 KO mice compared to that in BMDCs from WT mice (*p* < 0.001; Figure 5b). In addition, OVA-treated BMDCs (OVA-BMDCs) of Flt3 KO mice had significantly higher levels of IL-10 and IL-6, which are known as Th2- [36,37] and Th17-inducing cytokines [38,39], respectively (*p* < 0.001 and *p* < 0.01, respectively), but there were similar levels of IL-12, a Th1-inducing cytokine, compared to those of WT mice (Figure 5c). These results suggest that the Th2-inducing function of DCs was stronger in Flt3 KO mice than in WT mice, despite the decreased population of DCs. 

To confirm this, co-culture experiments using BMDCs and CD4^+^ T cells were performed. BMDCs from both WT and Flt3 KO mice efficiently induced CD4^+^ T cell proliferation (Figure 5d). CD4^+^ T cells cultured with OVA-BMDCs from Flt3 KO mice produced higher levels of IL-5 and IL-13 but lower levels of IL-17 compared to those from WT mice; the levels of IFN-γ tended to decrease (*p* < 0.001 for IL-5, IL-13, and IL-17; *p* = 0.400 for IFN-γ) (Figure 5e). Furthermore, the expression of T cell transcription factors was analyzed (Supplementary Figure S3). The GATA binding protein 3 (GATA3)/T-box expressed in T cells (T-bet) ratio was higher in CD4^+^ T cells cultured with OVA-BMDCs of Flt3 KO mice than in those cultured with OVA-BMDCs of WT mice (*p* < 0.01) (Figure 5e, Supplementary Figure S3a,b).

### 2.6. Adoptive Transfer of BMDCs from Flt3 KO Mice Exacerbates Asthma with More Marked Th2 Immune Responses Compared to That of BDMCs from WT Mice

**Figure 6:**
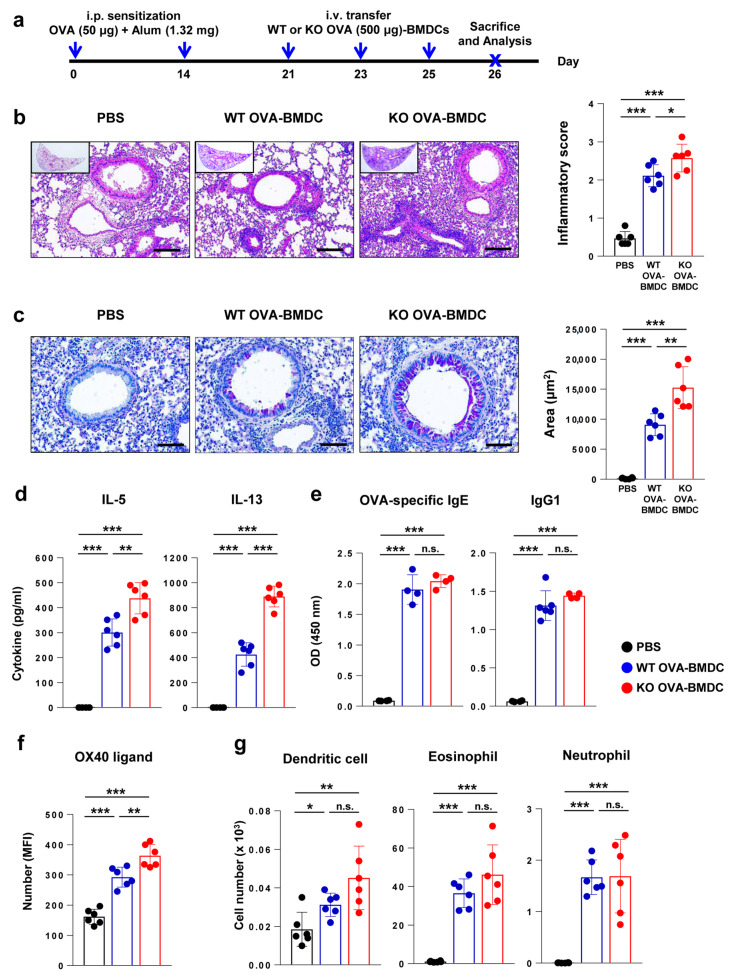
Comparison of allergic asthma phenotype and relevant immune responses following the adoptive transfer of bone marrow-derived dendritic cells (BMDCs) from wild-type (WT) and Flt3 knockout (KO) mice. (**a**) Experimental schematic protocol of allergic asthma induction through the intravenous transfer of ovalbumin (OVA)-loaded BMDCs. Arrows indicate the time points of procedures or sacrifice. i.p., intraperitoneal; i.v., intravenous. (**b**) Representative hematoxylin and eosin-stained sections (original magnification: ×100, magnified image of the ×10 figure in the left upper quadrant) and inflammatory score based on lung histology (scale bar: 100 μm). (**c**) Representative periodic acid-Schiff (PAS)-stained section (original magnification: ×200) and PAS-positive areas based on lung histology (scale bar: 50 μm) in mice administered BMDCs from WT and Flt3 KO mice. (**d**) Cytokines measured by ex vivo recall assays with OVA protein (10 μg/mL) in isolated mediastinal lymph node cells. (**e**) OVA-specific IgE and IgG1 in serum, as measured using ELISA. (**f**) OX40 ligand level in lung DCs, as measured by flow cytometry. (**g**) Immune cell population of BALF. Data are representative of two independent experiments with six mice/group in each experiment. The results are expressed as the mean ± standard deviation. One-way analysis of variance followed by Tukey’s multiple comparison tests was used to evaluate the significance of differences. * *p* < 0.05, ** *p* < 0.01, *** *p* < 0.001.

Furthermore, to confirm the Th2-inducing effect of BMDCs from Flt3 KO mice in vivo, we used an asthma model via the adoptive transfer of OVA-loaded BMDCs (OVA-BMDCs) (Figure 6a) [30]. OVA-BMDCs were checked for purity >90% and then injected intravenously (i.v.) into OVA-sensitized mice. Mice administered OVA-BMDCs from Flt3 KO mice developed increased inflammatory cell infiltration (*p* < 0.05; Figure 6b) and goblet cell hyperplasia compared to those administered OVA-BMDCs from WT mice (*p* < 0.01; Figure 6c). 

In addition, IL-5 and IL-13 levels from OVA-re-stimulated mediastinal lymph node cells (Figure 6d) as well as OX40L expression in lung cells (Figure 6f) were significantly increased in mice transferred with OVA-BMDCs from Flt3 KO mice compared to in mice transferred with OVA-BMDCs from WT mice (*p* < 0.001). Levels of serum OVA-specific IgE and IgG1 were not significantly different between the mice injected with OVA-BMDCs from Flt3 KO mice and those injected with OVA-BMDCs from WT mice (Figure 6e). There was no significant difference in immune cell populations of BALF between OVA-treated WT mice and OVA-treated Flt3 KO mice (Figure 6g). Collectively, these results confirm the enhanced Th2 induction ability of DCs from Flt3 KO mice compared to that in WT mice.

### 2.7. Flt3 KO Mice Exhibit Increased Expression of OX40L in DCs Indicating That Flt3 Signals Inhibit the Expression of OX40L

**Figure 7:**
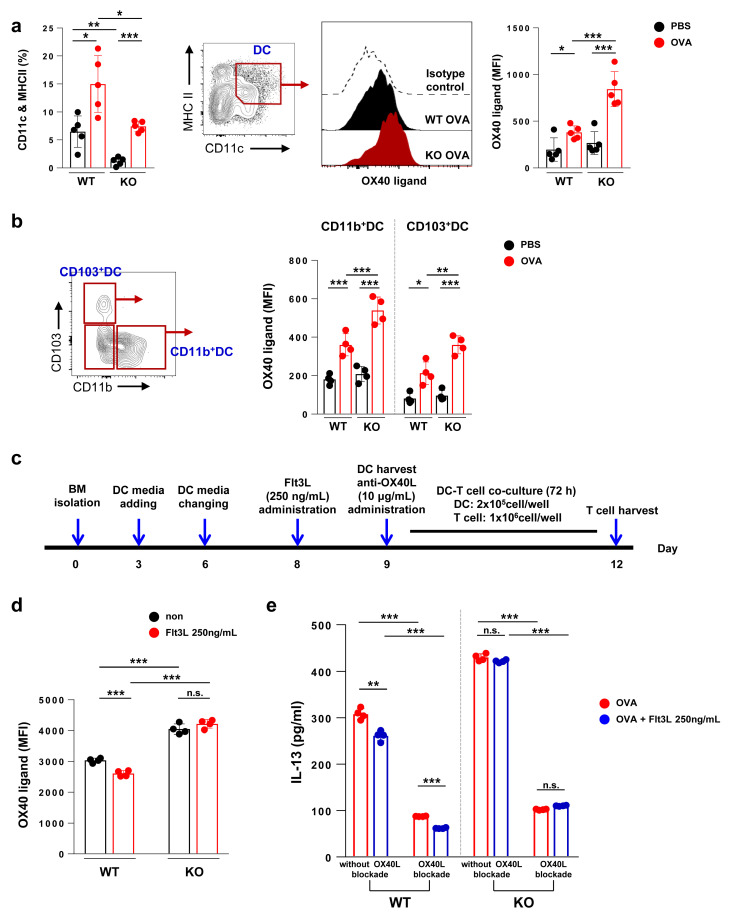
Expression of OX40 ligand (OX40L) in dendritic cells (DCs) from wild-type (WT) and Flt3 knockout (KO) mice. (**a**) Comparison of CD11c and MHC II double-positive cells (left panel). Gating strategy for investigating the OX40L level in lung DCs (center panel) and comparison of OX40L expression in ovalbumin (OVA)-treated WT and Flt3 KO mice (right panel). (**b**) Gating strategy for investigating CD11b^+^ DCs and CD103^+^ DCs (left panel). Comparison of OX40L expression in CD11b^+^ DCs and CD103^+^ DCs of OVA-treated WT and Flt3 KO mice (right panel). (**c**) Schematic protocol for the generation of bone marrow-derived dendritic cells (BMDCs) and their co-culture with CD4^+^ T cells from the spleens of OVA-specific OT-II Tg mice. Some cultures were supplemented with the Flt3 ligand (Flt3L; 250 ng/mL) or anti-OX40L (10 μg/mL). (**d**) Comparison of OX40L expression following Flt3L administration in BMDCs derived from WT and Flt3 KO mice. (**e**) Comparison of the IL-13 level in CD4^+^ T cells cultured with OVA-treated BMDCs from WT and Flt3KO mice following OX40L blockade and Flt3L administration. Data are representative of three independent experiments with four to five mice/group in each experiment. The results are expressed as the mean ± standard deviation. The significance of differences was analyzed using an unpaired Student’s t-test. * *p* < 0.05; ** *p* < 0.01; *** *p* < 0.001.

Lastly, to further investigate the stronger Th2-inducing function of DCs in Flt3 KO mice, we compared the functional properties of DCs between WT and Flt3 KO mice. The levels of OX40L, which promotes the Th2 immune responses [34,35], were significantly higher in the lung DCs of OVA-treated Flt3 KO mice than in those of OVA-treated WT mice (*p* < 0.01; Figure 7a). Since there was a major shift in the subset of lung DCs in Flt3 KO mice, we independently measured the OX40L levels in CD11b^+^ DCs and CD103^+^ DCs. As expected, OX40L expression was higher in CD11b^+^ DCs than in CD103^+^ DCs (*p* < 0.01 for OVA-treated WT mice; *p* < 0.001 for OVA-treated Flt3 KO mice; Figure 7b). These results suggest that the relative increase in CD11b^+^ DCs (unbalanced CD11b^+^ DCs/CD103^+^ DCs ratio) in Flt3 KO mice enhanced OX40L expression, resulting in exacerbated asthma.

Then, we hypothesized that the Flt3 signal could inhibit OX40L expression. To confirm this, we examined the BMDCs from WT and Flt3 KO mice following Flt3L administration and OX40L blockade (Figure 7c). As expected, Flt3L administration inhibited OX40L expression in the BMDCs of WT mice (*p* < 0.001; Figure 7d). However, this phenomenon was not observed in the BMDCs of Flt3 KO mice, which is probably due to the absence of the Flt3 receptor (Figure 7d). Since the IL-13 level was markedly increased in OVA-treated Flt3 KO mice (Figure 4a and Figure 5e, and Supplementary Figure S1), we verified whether this would also be affected by the Flt3L administration and OX40L blockade using the co-culture of BMDCs and CD4^+^ T cells. All experimental conditions of BMDCs from both WT and Flt3 KO mice efficiently induced CD4^+^ T cell proliferation (Supplementary Figure S4). Notably, the IL-13 level was significantly decreased in CD4^+^ T cells cultured with BMDCs with OX40L blockade compared to that in CD4^+^ T cells cultured with BMDCs without OX40L blockade (*p* < 0.001; Figure 7e). In addition, Flt3L administration reduced the IL-13 levels in CD4^+^ T cells cultured with BMDCs from WT mice; however, this was not observed in case of CD4^+^ T cells cultured with BMDCs from Flt3KO mice (Figure 7e). Taken together, these results confirm that the Flt3 signal downregulates OX40L expression.

## 3. Discussion

**Figure 8:**
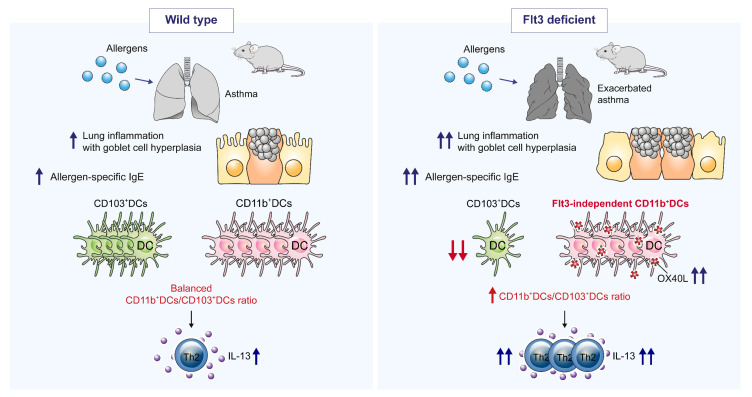
Schematic of changes in allergic asthma and the population of dendritic cells (DCs) associated with Flt3 (Fms-like tyrosine kinase 3) deficiency. Allergic asthma was sufficiently induced in Flt3 knockout (KO) mice, even though DCs, and particularly CD103^+^ DCs, were deficient. Upon allergen challenge, lung inflammation with goblet cell hyperplasia, serum allergen-specific IgE levels, and IL-13 in the lung were increased in Flt3 KO mice, compared to the case in WT mice. These findings were associated with OX40 ligand (OX40L) expression, which was inhibited by the Flt3 ligand. Thus, Flt3-independent CD11b^+^ DCs are the principal cause of exacerbated Th2 immune responses; the ratio of CD11b^+^ DCs to CD103^+^ DCs is important for allergic inflammation.

In the present study, allergic airway inflammation was sufficiently induced in Flt3 KO mice (Figure 1 and Figure 2), even though DCs, and particularly CD103^+^ DCs, were deficient (Figure 3). This was accompanied by an immunological shift toward the Th2 responses in Flt3 KO mice (Figure 1 and Figure 4). We found that DCs—independent of the Flt3 signal—were the major factor in advancing allergic asthma by directing the type 2 immune responses, which was confirmed through in vitro (Figure 5) and in vivo studies (Figure 6). Moreover, we showed that these findings were associated with OX40L expression, which was downregulated by Flt3L (Figure 7). These results are summarized in Figure 8.

Flt3L is essential for the development of DCs, and mice lacking Flt3L or Flt3 showed severe DC deficiency, which is more apparent in Flt3L KO mice than in Flt3 KO mice [21]. Interestingly, Flt3L treatment not only expands DC populations but also alleviates airway inflammation and AHR in mouse models of asthma [27,28]. Thus, it is important to evaluate the phenotypic and immunologic effects of Flt3 KO on allergic asthma.

In the lungs of Flt3 KO mice, total DCs were significantly decreased; notably, CD103^+^ DCs were nearly absent. CD11b^+^ DCs were also markedly decreased in the lungs of PBS-treated control groups. However, CD11b^+^ DCs were detected in the OVA-treated groups, accounting for most of the total DCs. Therefore, Flt3-independent DCs (mainly CD11b^+^ DCs of Flt3 KO mice) might have a superior Th2-inducing function as compared to Flt3-dependent DCs (CD103^+^ DCs), although this population was decreased in the lungs of Flt3 KO mice. CD103^+^ DCs could have a protective role during Th2-mediated immune responses. The ratio of CD11b^+^ DCs to CD103^+^ DCs is important for the initiation and development of allergic inflammation (Figure 3b).

These results correspond to those of previous studies suggesting that CD11b^+^ DCs are important for inducing allergic asthma and Th2 immunity [40,41]. In contrast, in Batf3-deficient mice, which lack lung CD103^+^ DCs, allergen challenge resulted in increases in Th2 and Th17 immune responses [42], indicating that CD103^+^ DCs promote airway tolerance by inducing Foxp3^+^ Treg expression [43] or Treg-independent IL-10 production [44]. Another study reported that Flt3L KO mice show no defects in the development of allergic airway inflammation, indicating that Flt3L-dependent DCs are not required for inducing airway inflammation or tolerance [45]. Therefore, the sufficient induction of asthmatic inflammation in Flt3 KO mice in the present study might be due to both the lack of CD103^+^ DCs and improved Th2-inducing functions of infiltrated CD11b^+^ DCs.

OX40L and GM-CSF are thought to be responsible for enhanced Th2 induction in the absence of Flt3 signaling. OX40L, expressed on the surfaces of activated DCs, interacts with OX40 on T cells and promotes commitment toward the Th2 lineage [34]. OX40L expression is increased in patients with asthma and mouse models of asthma; consequently, blocking OX40/OX40L mediated the inhibition of Th2 responses [35,46,47]. GM-CSF and Flt3L are important growth factors for DC development [48,49]. GM-CSF has a critical role in eosinophil survival and asthma development [41,50]. GM-CSF promotes type 2 immunity to allergen and increases the susceptibility to allergic asthma through the GM-CSF/IL-33/OX40L pathway [51].

In the present study, the level of OX40L was significantly higher in DCs from Flt3 KO mice than in those from WT mice in both in vitro (Figure 5b) and in vivo studies (Figure 7a), as evidenced by the adoptive transfer of OVA-loaded BMDCs (Figure 6e). Since CD11b^+^ DCs exhibited higher OX40L expression than CD103^+^ DCs (Figure 7b), the relative increase in infiltrated CD11b^+^ DCs, i.e., the unbalanced CD11b^+^ DCs/CD103^+^ DCs ratio in Flt3 KO mice, could be the cause of increased OX40L expression, thus resulting in exacerbated Th2 immune responses. Furthermore, in the OX40L blockade experiment, we verified that the Flt3 signal inhibited OX40L expression and affected IL-13 levels (Figure 7d,e). The level of IL-13, Th2 cytokine, was significantly elevated in Flt3 KO mice than in WT mice (Figure 4a and Figure 5e, and Supplementary Figure S1). This is supported by increased serum allergen-specific IgE levels and goblet cell hyperplasia in Flt3 KO mice compared to those in WT mice because IL-13 can cause mucus hypersecretion and is involved in the production of IgE [52,53]. Collectively, these findings are important because they provide evidence that Flt3 is required to control type 2 allergic inflammation.

There are some limitations in the present study. First of all, the subset of CD11b^+^ DCs in Flt3-deficient condition should be further analyzed by subdividing them into moDCs and CD11b^+^ cDCs. The propensity for Th2 immune responses in Flt3 KO mice can be due to the increased number of moDCs that surpasses CD11b^+^ cDCs or altered function of moDCs and CD11b^+^ cDCs. This question could be clarified using markers for moDCs, such as Ly6C, Mar-1, and CCR2 [40,54]. We are planning further research on the subset of CD11b^+^ DCs in an Flt3-deficient condition including the measurement of OX40L. Moreover, we have yet to investigate the expression of IL-13^+^ CD4^+^ T cells in the lung to complement cytokine profiles observed in lung cells (Figure 4a). Furthermore, more investigation is warranted to define the different expression pattern between Th2 cytokines in Flt3 KO mice (Figure 4a). In addition, the asthma model using OVA is not physiologically relevant in humans. Further studies investigating the asthma model using other allergens such as house dust mites are needed [55].

Despite these limitations, the present study could have a significant impact on understanding the immunologic mechanism of asthma. To the best of our knowledge, this is the first study to characterize and analyze BMDCs from Flt3 KO mice cultured with GM-CSF. In addition, we examined the phenotype and immune cell population, as well as detailed immune responses, associated with Flt3 deficiency in allergic asthma. We confirmed these results with in vitro and in vivo studies using an asthma model prepared by the adoptive transfer of OVA-loaded BMDCs. Furthermore, we demonstrated that the Flt3 signaling inhibited the expression of OX40L.

## 4. Materials and Methods

### 4.1. Animals

C57BL/6J female mice, 6–7 weeks of age, were purchased from Japan SLC, Inc. (Shizuoka, Japan) and maintained under specific pathogen-free conditions. Age- and sex-matched Flt3 KO mice on the same genetic background with C57BL/6J mice were kindly provided by Dr. J.-H. Choi (Hanyang University, Seoul, Korea). The use of animals in this study was approved by the Ethics Committee and Institutional Animal Care and Use Committee of Yonsei University Health System (Permit no.: 2017-0342, approved at 30 March 2017), and the study was conducted according to international guidelines (ARRIVE) on animal experiments.

### 4.2. Mouse Models of Allergic Asthma

Allergic asthma was induced using a conventional method via intraperitoneal sensitization followed by intranasal challenge with OVA, as described previously with slight modifications [56]. Mice were sensitized on days 0 and 14 by an intraperitoneal (i.p.) injection of 50 μg of OVA (grade V; Sigma-Aldrich, St. Louis, MO, USA) emulsified in 1.32 mg of aluminum hydroxide gel (alum) (Sigma-Aldrich) in a total volume of 200 μL. PBS-treated mice were used as the control group. The anesthetized mice were challenged intranasally (i.n.) with 150 μg of OVA in 30 μL of PBS for 5 consecutive days starting from day 21. Mice were analyzed at 24 h following the last challenge.

### 4.3. Generation of Bone Marrow-Derived Dendritic Cells (BMDCs) and Adoptive Transfer of OVA-Loaded BMDCs to Induce Allergic Asthma

BMDCs were generated from bone marrow (BM) using conventional methods [57,58]. Furthermore, we established an asthma model via the intravenous (i.v.) transfer of OVA-loaded BMDCs [59]. Following intraperitoneal OVA sensitization on days 0 and 14, 1 × 10^7^ OVA (500 μg)-loaded BMDCs in a total volume of 200 μL were injected i.v. (via the tail vein) into non-anesthetized mice on days 21, 23, and 25. Mice were analyzed 24 h following the last transfer.

### 4.4. DC-T Cell Co-Culture and T Cell Proliferation Assay

CD4^+^ T cells were isolated from the spleen of OVA-specific OT-II Tg mice using magnetic bead purification (MACS; Miltenyi Biotec, Bergisch Gladbach, Germany). The cells (1 × 10^6^ cells/well) were followed by co-culture with BMDCs (2 × 10^5^ cells/well) in the presence or absence of OVA (10 μg/mL) for 72 h [60]. T cell proliferation was assessed by flow cytometry using violet proliferation dye 450 (VPD450) (BD Biosciences, San Jose, CA, USA) [61].

### 4.5. Histological Assessment of Lung Tissue

Paraffin-embedded lung tissue sections were stained with hematoxylin and eosin or PAS solution. Lung inflammation and goblet cell hyperplasia were graded using a previously reported semiquantitative scoring system [62,63].

### 4.6. Enzyme-Linked Immunosorbent Assay (ELISA) for Allergen-Specific Antibodies

Serum allergen-specific antibodies (Abs) were quantified via sandwich ELISA as described previously with minor modifications [64].

### 4.7. Analysis of Airway Hyperresponsiveness (AHR)

AHR to inhaled methacholine (Sigma-Aldrich, St. Louis, MO, USA) was evaluated with forced oscillation measurements using the Flexivent system (SCIREQ, Montreal, QC, Canada) as described previously [65].

### 4.8. Flow Cytometric Analysis and Intracellular Cytokine Staining

Single cells from the lungs, spleens, and mediastinal lymph nodes were stained with fluorochrome-conjugated antibodies to examine myeloid cells and then analyzed using BD LSR II Fortessa flow cytometry (BD Biosciences) and FlowJo software (Tree Star, Ashland, OR, USA). Intracellular cytokines of CD4^+^ T cells were measured by re-stimulating the cells with OVA protein (10 μg/mL), followed by intracellular staining with fluorochrome-conjugated antibodies.

### 4.9. Statistical Analysis

All data are representative of at least two or three independent experiments, with 4–8 mice per group in each experiment. Differences between two groups were analyzed using an unpaired Student’s *t*-test. One-way analysis of variance followed by Tukey’s multiple comparison tests was used to analyze data with more than two groups. Statistical analyses were conducted using Prism (GraphPad Software version 8, San Diego, CA, USA). 

Details of the other methods employed in the present study are included in the Supplementary Materials.

## 5. Conclusions

In summary, upon allergen challenge, the hyperplasia of goblet cells and sufficient infiltration of inflammatory cells were observed in the lung of Flt3 KO mice. Serum allergen-specific IgE levels and IL-13 and OX40L in the lung were significantly elevated in Flt3 KO mice compared to those in WT mice. Moreover, mice administered OVA-BMDCs from Flt3 KO mice showed more severe allergic airway inflammation compared to those administered OVA-BMDCs from WT mice. These results demonstrate that Flt3 KO mice have the propensity for exhibiting the Th2 responses, even though DCs, and particularly CD103^+^ DCs, were deficient. Therefore, Flt3-independent CD11b^+^ DCs are the principal cause of exacerbated Th2 immune responses; the ratio of CD11b^+^ DCs to CD103^+^ DCs is important for allergic inflammation. Additionally, the Flt3L was found to inhibit OX40L expression, suggesting that Flt3 and relevant signaling are required to control type 2 allergic inflammation. These results will contribute to studies on the pathophysiology of allergic asthma based on DCs and help identify anti-inflammatory therapies for asthma.

## Supplementary Materials

Supplementary materials can be found at https://www.mdpi.com/1422-0067/21/24/9508/s1.

## Abbreviations

AHR	airway hyperresponsiveness
BAL	bronchoalveolar lavage
BDCA-3	blood dendritic cell antigen 3
BMDC	bone marrow-derived dendritic cell
DC	dendritic cell
Flt3	Fms-like tyrosine kinase 3
Flt3L	Fms-like tyrosine kinase 3 ligand
GM-CSF	granulocyte-macrophage colony-stimulating factor
Ig	immunoglobulin
KO	knockout
OVA	Ovalbumin
OX40L	OX40 ligand
PAS	periodic acid-Schiff
PBS	phosphate-buffered saline
Treg	regulatory T cell
WT	wild-type
