# Supplementary material for: Fms-Like Tyrosine Kinase 3-Independent Dendritic Cells Are Major Mediators of Th2 Immune Responses in Allergen-Induced Asthmatic Mice
Source: Int J Mol Sci. 2020 Dec 14;21(24):9508. doi: 10.3390/ijms21249508 (PMC7765069; doi:10.3390/ijms21249508)
Supplement: Supplementary file 1 [file ijms-21-09508-s001.pdf]

Supplementary Materials

Fms-Like Tyrosine Kinase 3-Independent Dendritic Cells Are Major Mediators of Th2 Immune Responses in Allergen-Induced Asthmatic Mice

Sang Chul Park, Dahee Shim, Hongmin Kim, Yeeun Bak, Da Yeon Choi, Joo-Heon Yoon, Chang-Hoon Kim and Sung Jae Shin

1. Supplementary Methods

1.1. Histological Assessment of Lung Tissue

Lung tissue was fixed in 10% neutral-buffered formalin and embedded in paraffin. Paraffin-embedded tissue sections were stained with hematoxylin and eosin or periodic acid-Schiff (PAS) solution. Lung inflammation and goblet cell hyperplasia were graded using a previously reported semiquantitative scoring system [1,2]. To grade inflammatory cell infiltration, peribronchial cell counts were determined based on a five-point grading system for the following features: 0, normal; 1, few cells; 2, a ring of inflammatory cells one-cell layer deep; 3, a ring of inflammatory cells > 2–4 cells deep; 4, a ring of inflammatory cells > 4 cells deep. Eight fields were counted for each section, and the mean score was calculated from five to seven mice per group. To quantify airway goblet cells, PAS-positive areas were measured in pixels and converted into μm2 using ImageJ software (version 1.51j8; National Institutes of Health, Bethesda, MD, USA). In some experiments, the following five-point grading system was used: 0, <5 % PAS-positive cells; 1, 5–25%; 2, 25–50%; 3, 50–75%; and 4, >75%. Eight fields were counted for each section, and the mean area or mean score was calculated from five to seven mice per group. Analyses were performed in a blinded manner, and slides were presented in random order for each examination.

1.2. Analysis of Airway Hyperresponsiveness (AHR)

AHR to inhaled methacholine (Sigma-Aldrich, St. Louis, MO, USA) was measured in forced oscillation measurements using the Flexivent system (SCIREQ, Montreal, QC, Canada) at 24 h following the last OVA challenge, as described previously [3]. Briefly, anesthetized and paralyzed mice were tracheotomized, cannulated, and mechanically ventilated at 2.5 Hz. Airway resistance and elastance were determined in response to increasing doses of aerosolized methacholine (0, 1.56, 3.125, 6.25, 12.5, 25, and 50 mg/mL).

1.3. Analysis of Cellular Composition in Bronchoalveolar Lavage Fluid (BALF)

To harvest BALF, the lungs were lavaged with 1 mL of PBS containing 0.5 mM EDTA via a 1-mL syringe inserted in the cannula. The BALF was harvested by centrifugation at 2000 rpm for 3 min at 4 °C. Thereafter, the cell pellet was resuspended in 200 μL of FACS buffer and probed with antibodies for flow cytometry analysis to examine total and differential cell counts.

1.4. Single-Cell Preparation

To harvest single cells from tissues, the lungs, spleens, and mediastinal lymph nodes (mLN) were harvested and incubated in RPMI 1640 digestion media (10% fetal bovine serum, 0.1% collagenase type II (Worthington Lakewood, NJ, USA), 1 mM MgCl2, and 1 mM CaCl2) at 37 °C for 30 min. Single-cell suspensions were then filtered through a 40-μm cell nylon mesh cell strainer, treated with RBC lysis buffer (Sigma-Aldrich) for 5 min, and washed twice with RPMI 1640 medium

supplemented with 2% fetal bovine serum. Lung, spleen, and mLN cells were seeded onto 96-well round bottom plates at densities of 1×10^6, 2×10^6, and 2×10^5 cells/well, respectively.

1.5. Flow Cytometric Analysis and Intracellular Cytokine Staining

To analyze myeloid cells, single-cell suspensions were initially blocked with Fc Block (anti-CD16/32; eBioscience, San Diego, CA, USA) for 15 min at 4 °C and then stained with fluorochrome-conjugated anti-CD3e (clone: 17A2), anti-CD11b (clone: M1/70), anti-CD103 (clone: 2E7), anti-F4/80 (clone: BM8) (eBioscience), anti-CD4 (clone: BM8), anti-Ly6G (clone: 1A8), anti-Siglec-F (clone: E50-2440) (BD Biosciences, San Jose, CA, USA), and anti-MHC II (clone: M5/114.15.2), anti-CD11c (clone: N418), anti-CD19 (clone: 6D5), and anti-CD64 (clone: X54-5/7.1) (Biolegend, San Diego, CA, USA) antibodies for 30 min at 4 °C. Cells stained with appropriate isotype-matched antibodies were used as negative controls.

T-cell transcription factor and regulatory T cell (Treg) expression was analyzed by staining using fluorochrome-conjugated anti-CD3e (clone: 17A2) (eBioscience), anti-CD4 (clone: BM8), and anti-CD25 (clone: 7D4) (BD bioscience) antibodies, followed by permeabilization with a fixation/permeabilization kit (eBioscience) in accordance with the manufacturer's instructions and intracellular staining with anti-T-bet (clone: 4B10), anti-GATA3 (clone: TWAJ), anti-RORγt (clone: AFKJS-9), and anti-Foxp3 (clone: NRRF-30) (eBioscience) antibodies.

To re-stimulate the cells with the antigen, lung (1×10^6 cells/well) and spleen cells (2×10^6 cells/well) were re-stimulated with OVA protein (10 μg/mL) at 37 °C. Intracellular cytokines of $CD4^+$ T cells were quantified after 2 h of incubation with the antigen, 4 h of incubation with Golgistop (eBioscience), and permeabilization, which was followed by intracellular staining with fluorochrome-conjugated anti-IFN-γ (clone: XMG1.2), anti-IL-4 (clone: 11B11), anti-IL-5 (clone: TRFK5), anti-IL-10 (clone: JES5-16E3), and anti-IL-17 (clone: B8KN8R) (eBioscience) antibodies. The stained cells were analyzed using a BD LSR II Fortessa flow cytometer (BD Biosciences) and FlowJo software (Tree Star, Inc., Ashland, OR, USA). The flow cytometry results were analyzed using t-distributed stochastic neighbor embedding (t-SNE), a dimensionality reduction method [4].

To determine cytokine levels using an ex vivo recall assay, the supernatants were harvested 72 h after incubation with the antigen, and cytokine production was analyzed using enzyme-linked immunosorbent assay (ELISA).

1.6. ELISA for Antigen-Specific Antibodies

Serum antigen-specific antibodies were quantified using sandwich ELISA, as described previously with minor modifications [5]. Plates were coated with OVA (1 mg/mL). Serum dilutions were 1:50 for OVA-specific IgE, 1:100 for OVA-specific IgG2b and IgG2c, 1:5000 for OVA-specific IgG2a, and 1:10,000 for OVA-specific IgG1. Biotinylated (biotin) anti-mouse IgE (clone: R35-118), biotin anti-mouse IgG1 (clone: A85-1), biotin anti-mouse IgG2a (clone: R19-15), biotin anti-mouse IgG2b (clone: R12-3) (BD Biosciences), and biotin anti-mouse IgG2c (SouthernBiotech, Birmingham, AL, USA) were used, followed by incubation with horseradish peroxidase-conjugated streptavidin.

1.7. Generation of Bone Marrow-Derived Dendritic Cells (BMDCs)

BMDCs were generated from bone marrow (BM) using conventional methods [6,7]. Whole BM cells isolated from C57BL/6J or Fms-like tyrosine kinase 3 (Flt3) knockout (KO) mice were cultured in RPMI 1640 medium supplemented with 100 U/mL of penicillin/streptomycin (Lonza, Basel, Switzerland), 10% fetal bovine serum (Lonza), 50 μM mercaptoethanol (Lonza), and 20 ng/mL of GM-CSF plus 5 ng/mL of IL-4 at 37 °C in the presence of 5 % CO2. On days 3 and 6, the medium was refreshed. On day 8, the purity of CD11c-positive non-adherent cells was confirmed to be >90 %, and the cells were harvested. Next, equal numbers (1×10^6 cells/mL) of cells from WT and Flt3 KO mice were incubated in 24-well plates in the presence of OVA protein (10 μg/mL for in vitro experiments or 500 μg/mL for adoptive transfer to mice) for 24 h. In some experiments, Flt3 ligand (250 ng/mL)

(eBioscience) was administered. On day 9, OVA-loaded BMDCs were harvested. Cytokine levels were measured in the culture supernatant.

1.8. DC-T Cell Co-culture and T Cell Proliferation Assay

On day 9, the expression of cell surface protein and cytokine of OVA-loaded BMDCs were examined using flow cytometry and ELISA, respectively. $CD4^+$ T cells were isolated from the spleen of OVA-specific OT-II Tg mice using magnetic bead purification (MACS; Miltenyi Biotec, Bergisch Gladbach, Germany). In total, BMDCs (2×10^5 cells/well) were seeded onto a round-bottomed 96-well plate and cultured with $CD4^+$ T cells (1×10^6 cells/well) at a 1:5 DC:T cell ratio in the presence or absence of OVA (10 μg/mL) for 72 h [8]. In some experiments, anti-OX40L (10 μg/mL) (R&D Systems, Minneapolis, MN, USA) was administered for 30 min at 37 °C and then used in co-culture with $CD4^+$ T cells [9]. The supernatants were harvested, and cytokines were quantified using ELISA. Harvested cells were examined for transcription factors of T cells, including T-bet, GATA3, RORγt, and Foxp3 for Th1, Th2, Th17, and Treg cells, respectively, using flow cytometry.

T cell proliferation was assessed by flow cytometry using violet proliferation dye 450 (VPD450) (BD Biosciences) in accordance with the manufacturer's instructions [10]. Briefly, isolated $CD4^+$ T cells were prepared as a single-cell suspension at 1×10^6 cells/mL in PBS. For every 1 mL of the cell suspension, 1 μL of 1 mM VPD450 stock solution was added to yield a final VPD450 concentration of 1 μM. Following incubation in a water bath at 37 °C for 15 min, 10 mL of complete medium with 10 % fetal bovine serum was added, followed by centrifugation, elimination of the supernatant, resuspension of the pellet, and cell culture. Proliferative index was calculated from the violet fluorescence histograms by using the following formula, as previously reported [10].

2. Supplementary Figures

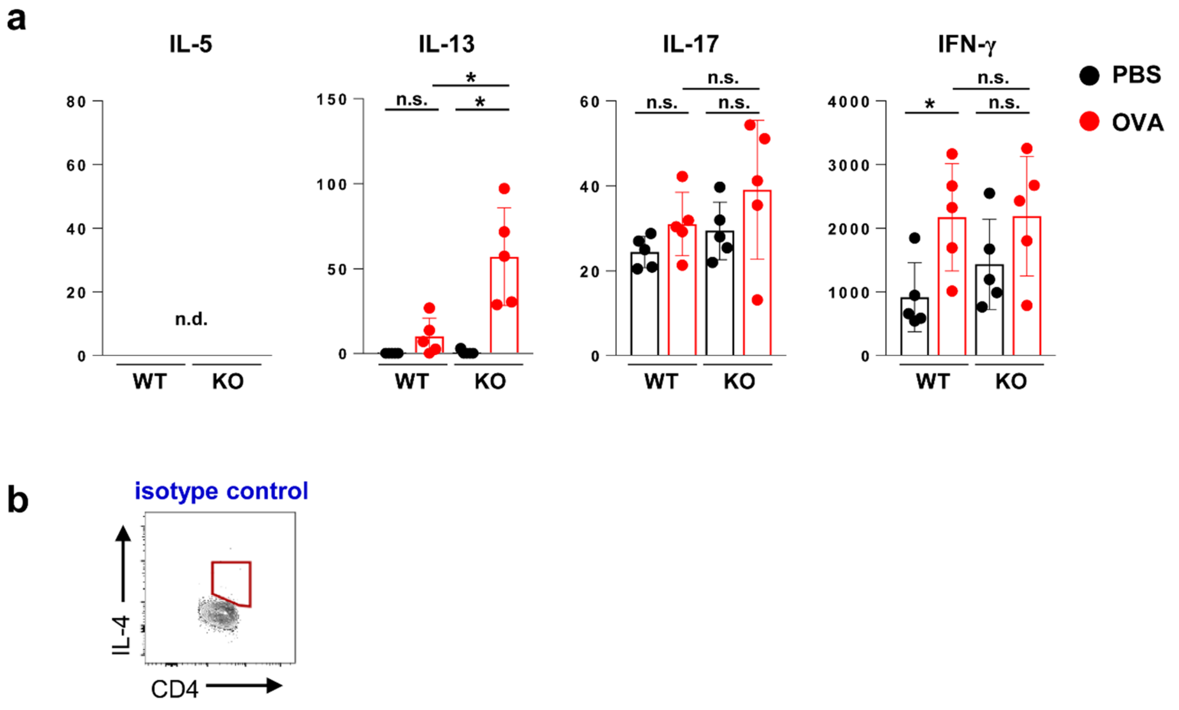

Figure S1. Cytokine profiles and analysis of T cell subpopulations in wild-type (WT) and Flt3 knockout (KO) mice. (**a**) Cytokines were measured using ex vivo recall assays with ovalbumin (OVA) protein (10 μg/mL) in isolated spleen cells. (**b**) Representative flow cytometry plots for isotype control of IL-4^+ $CD4^+$ T cells in the lung. Data are representative of three independent experiments with 5 mice/group in each experiment. The results are expressed as the mean ± standard deviation. The significance of differences was analyzed using an unpaired Student's *t*-test. * $p < 0.05$, ** $p < 0.01$, *** $p < 0.001$. n.d., not detected.

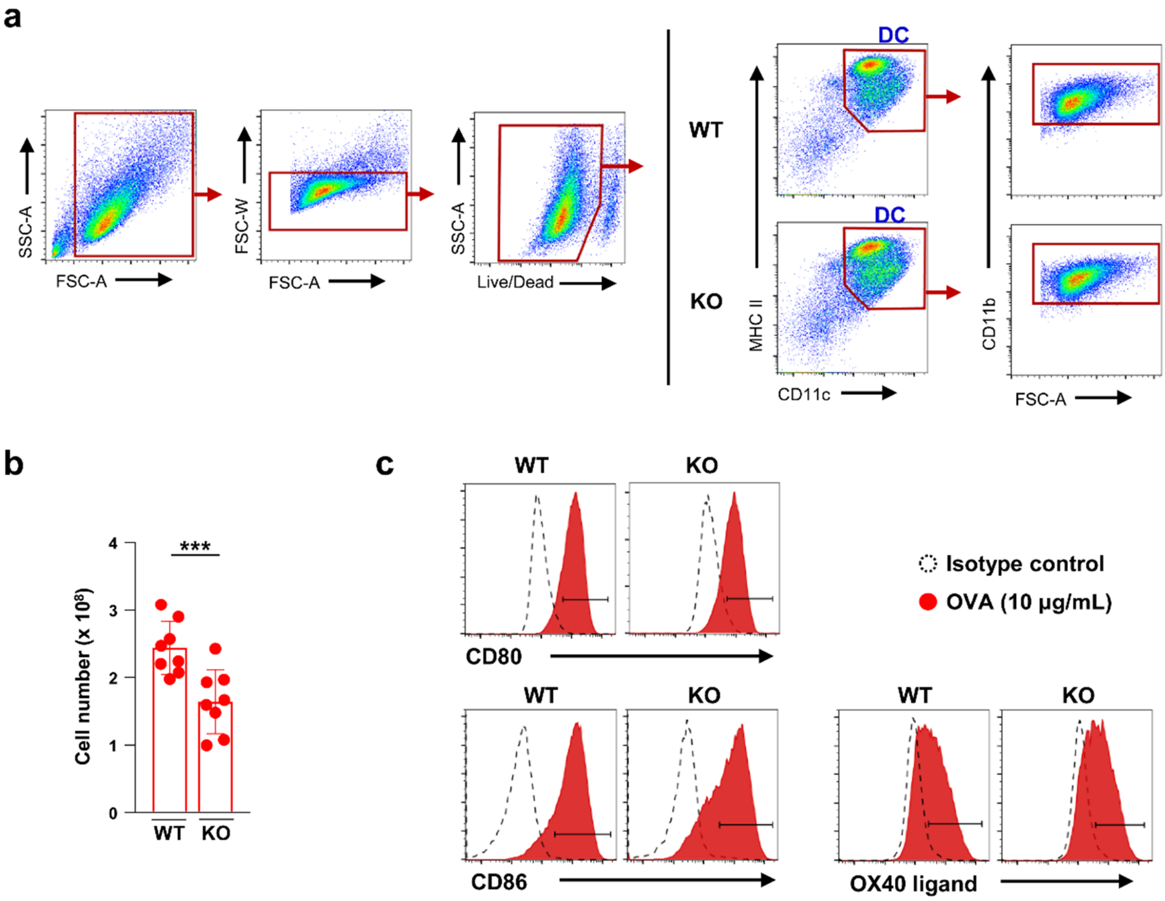

Figure S2. Analysis of bone marrow-derived dendritic cells (BMDCs). (**a**) Representative flow cytometry plots for BMDCs. CD11c and MHC II double-positive cells were gated from single cells. (**b**) The number of harvested BMDCs. (**c**) Representative flow cytometry plots for surface molecules of BMDCs in wild-type (WT) and Flt3 knockout (KO) mice. Data are representative of three independent experiments with 8 mice/group in b and 5 mice/group in c in each experiment. The results are expressed as the mean ± standard deviation. The significance of differences was analyzed using an unpaired Student's *t*-test. *** $p < 0.001$.

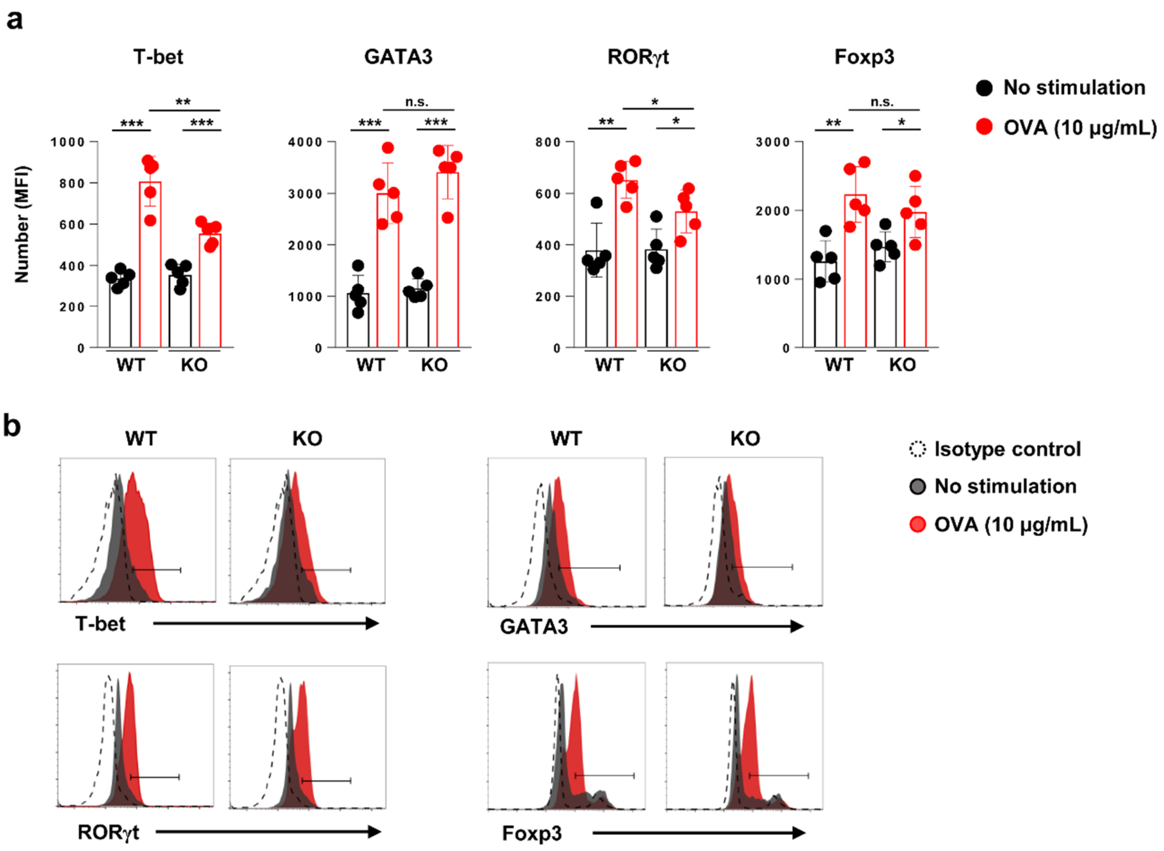

Figure S3. T cell transcription factors examined in CD4⁺ T cells cultured with bone marrow-derived dendritic cells (BMDCs). (**a**) Expression level of T cell transcription factors, as measured by flow cytometry. (**b**) Representative flow cytometry plots for T cell transcription factors in wild-type (WT) and Flt3 knockout (KO) mice. Data are representative of three independent experiments with 5 mice/group in each experiment. The results are expressed as the mean ± standard deviation. The significance of differences was analyzed using an unpaired Student's t-test. * $p < 0.05$, ** $p < 0.01$, *** $p < 0.001$.

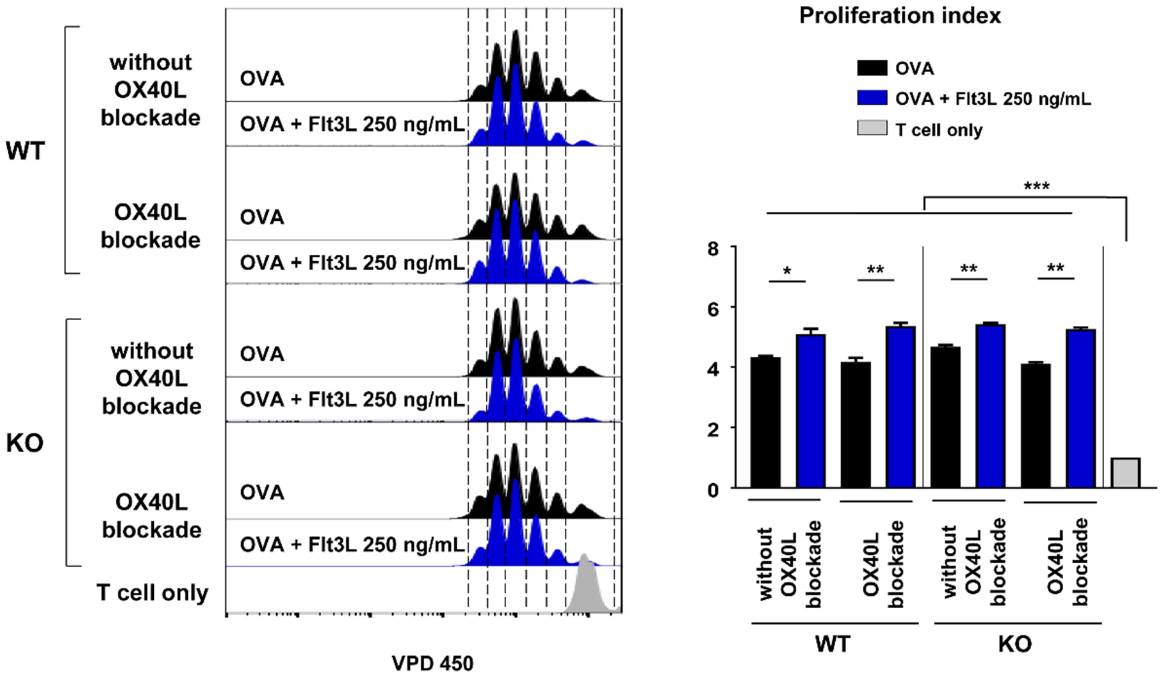

Figure S4. T cell proliferation assessed in CD4⁺ T cells cultured with ovalbumin (OVA)-treated bone marrow-derived dendritic cells (BMDCs) from wild-type (WT) and Flt3 knockout (KO) mice

following OX40 ligand blockade and Flt3 ligand administration. Data are representative of three independent experiments with 4 mice/group in each experiment. The results are expressed as the mean ± standard deviation. The significance of differences was analyzed using an unpaired Student's t-test. * $p < 0.05$; ** $p < 0.01$; *** $p < 0.001$.

References

1. Ozdoganoglu, T.; Songu, M. The burden of allergic rhinitis and asthma. *Ther. Adv. Respir. Dis.* **2012**, *6*, 11–23, doi: 10.1177/1753465811431975.
2. Yoshioka, M.; Sagara, H.; Takahashi, F.; Harada, N.; Nishio, K.; Mori, A.; Ushio, H.; Shimizu, K.; Okada, T.; Ota, M.; et al. Role of multidrug resistance-associated protein 1 in the pathogenesis of allergic airway inflammation. *Am. J. Physiol. Lung Cell Mol. Physiol.* **2009**, *296*, L30–L36, doi:10.1152/ajplung.00026.2008.
3. Saglani, S.; Gregory, L.G.; Manghera, A.K.; Branchett, W.J.; Uwadiae, F.; Entwistle, L.J.; Oliver, R.A.; Vasiliou, J.E.; Sherburn, R.; Lui, S.; et al. Inception of early-life allergen-induced airway hyperresponsiveness is reliant on IL-13(+)CD4(+) T cells. *Sci Immunol* **2018**, *3*, doi:10.1126/sciimmunol.aan4128.
4. Amir, E.-a.D.; Davis, K.L.; Tadmor, M.D.; Simonds, E.F.; Levine, J.H.; Bendall, S.C.; Shenfeld, D.K.; Krishnaswamy, S.; Nolan, G.P.; Pe'er, D. viSNE enables visualization of high dimensional single-cell data and reveals phenotypic heterogeneity of leukemia. *Nat. Biotechnol.* **2013**, *31*, 545, doi:10.1038/nbt.2594.
5. Kamijo, S.; Takeda, H.; Tokura, T.; Suzuki, M.; Inui, K.; Hara, M.; Matsuda, H.; Matsuda, A.; Oboki, K.; Ohno, T.; et al. IL-33-mediated innate response and adaptive immune cells contribute to maximum responses of protease allergen-induced allergic airway inflammation. *J. Immunol.* **2013**, *190*, 4489–4499, doi:10.4049/jimmunol.1201212.
6. Jang, A.R.; Kim, G.; Hong, J.J.; Kang, S.M.; Shin, S.J.; Park, J.H. Mycobacterium tuberculosis ESAT6 Drives the Activation and Maturation of Bone Marrow-Derived Dendritic Cells via TLR4-Mediated Signaling. *Immune Netw.* **2019**, *19*, e13, doi:10.4110/in.2019.19.e13.
7. Kim, J.S.; Kim, W.S.; Choi, H.G.; Jang, B.; Lee, K.; Park, J.H.; Kim, H.J.; Cho, S.N.; Shin, S.J. Mycobacterium tuberculosis RpfB drives Th1-type T cell immunity via a TLR4-dependent activation of dendritic cells. *J. Leukoc. Biol.* **2013**, *94*, 733–749, doi:10.1189/jlb.0912435.
8. Kwon, K.W.; Choi, H.H.; Han, S.J.; Kim, J.S.; Kim, W.S.; Kim, H.; Kim, L.H.; Kang, S.M.; Park, J.; Shin, S.J. Vaccine efficacy of a Mycobacterium tuberculosis Beijing-specific proline-glutamic acid (PE) antigen against highly virulent outbreak isolates. *FASEB J.* **2019**, *33*, 6483–6496, doi:10.1096/fj.201802604R.
9. Gopisetty, A.; Bhattacharya, P.; Haddad, C.; Bruno, J.C., Jr.; Vasu, C.; Miele, L.; Prabhakar, B.S. OX40L/Jagged1 cosignaling by GM-CSF-induced bone marrow-derived dendritic cells is required for the expansion of functional regulatory T cells. *J. Immunol.* **2013**, *190*, 5516–5525, doi:10.4049/jimmunol.1202298.
10. Santos, M.G.; Almeida, V.G.; Avelar-Freitas, B.A.; Grael, C.F.F.; Gregório, L.E.; Pereira, W.F.; Brito-Melo, G.E.A. Phytochemical screening of the dichloromethane-ethanolic extract of Eriosema campestre var. macrophylum roots and its antiproliferative effect on human peripheral blood lymphocytes. *Rev. Bras. Farmacogn.* **2016**, *26*, 464–470, doi:doi.org/10.1016/j.bjp.2015.08.009.
